# Mirabegron for medical expulsive therapy of ureteral stones: a systematic review and meta-analysis

**DOI:** 10.3389/fmed.2023.1280487

**Published:** 2024-01-05

**Authors:** Haifeng Song, Lei Liang, Hui Liu, Yubao Liu, Weiguo Hu, Gang Zhang, Bo Xiao, Meng Fu, Jianxing Li

**Affiliations:** Department of Urology, Beijing Tsinghua Changgung Hospital, School of Clinical Medicine, Tsinghua University, Beijing, China

**Keywords:** mirabegron, ureteral stone, medical expulsive therapy, meta-analysis, β3-adrenergic receptor

## Abstract

**Objective:**

To systematically review and quantitively evaluate the efficacy and safety of mirabegron as a medical expulsive therapy for ureteral stones.

**Methods:**

We performed an extensive search of the EMBASE and PubMed databases for studies examining the use of mirabegron as a medical expulsive therapy for ureteral stones. The primary outcome measure assessed was the stone expulsion rate (SER), while the secondary outcomes evaluated were the stone expulsion interval (SEI) and the occurrence of pain episodes during follow-up. Risk ratios (RRs) and mean differences (MDs) with their respective 95% CIs were calculated.

**Results:**

We included a total of seven studies involving 728 participants. Our analysis revealed a significant increase in the stone expulsion rate (SER) with mirabegron (RR = 1.40; 95% CI = 1.17–1.67; *p* < 0.001) and a reduction in the frequency of pain episodes (MD = −0.80; 95% CI = −0.39 to −0.21; *p* = 0.008) compared to the control group. No significant difference was found in SEI between the two groups (MD = −3.04; 95% CI = −6.33 to 0.25; *p* = 0.07). Subgroup analysis revealed that the increased SER was significant for distal ureteral stones, but not for proximal and middle ureter stones. Compared to tamsulosin or silodosin, mirabegron showed no significant difference in SER, SEI, or pain episode frequency. The adverse effects of mirabegron were relatively rare and mild.

**Conclusion:**

Mirabegron appears to be a promising candidate for the MET of distal ureteral stones rather than proximal and middle ureteral stones, as it significantly increases SER and reduces pain episode frequency. Further well-designed randomised controlled trials are needed to validate and affirm these findings.

**Systematic Review Registration:**

PROSPERO (CRD42022341603).

## Introduction

Urolithiasis is a common disease of the human urinary system and imposes a substantial burden on the healthcare system. The reported incidence rate varies from 1% to 10% worldwide (1). Stones within the ureters can cause severe pain and may lead to complications such as acute kidney injury, infections, and septic shock if left untreated, posing a significant threat to patient health and placing an economic burden on patients (2). The current primary treatment options for ureteral stones are medical expulsion therapy (MET), extracorporeal shockwave lithotripsy (ESWL), and endoscopic surgery. MET is recommended as a treatment option for distal ureteral stones measuring 5–10 mm in most clinical guidelines (3). MET promotes relaxation of smooth muscles in the urinary tract, thereby facilitating the passage of stones. The most widely recommended and used medications for this purpose are α-adrenergic antagonists, such as tamsulosin (4, 5).

Mirabegron, a β3-adrenergic receptor (β3-AR) agonist, alleviates overactive bladder symptoms by inducing bladder smooth muscle relaxation (6–8). Some studies have suggested that β3-AR is also expressed in the smooth muscle and urothelium of the human ureter (6, 9). Recent studies revealed that mirabegron has potential applications in the MET of ureteral stones, providing a novel alternative to traditional medications (10–17). However, the current evidence remains controversial and has not been comprehensively evaluated. This study aimed to summarise existing studies that systematically evaluated the effectiveness and safety of mirabegron in the MET of ureteral stones.

## Methods

The review protocol was registered on PROSPERO (CRD42022341603) in accordance with the Preferred Reporting Items for Systematic Reviews and Meta-Analyses (PRISMA) guidelines (18).

### Search strategy and study selection

The EMBASE and PubMed databases were systematically searched in order to identify all eligible clinical studies published prior to February 2023 with no language limitation. MeSH terms and keywords (mirabegron, β3-adrenergic receptor agonists, ureteral stones, and medical expulsive therapy) were utilized to search for related articles in the databases (Supplementary material).

**Figure 1:**
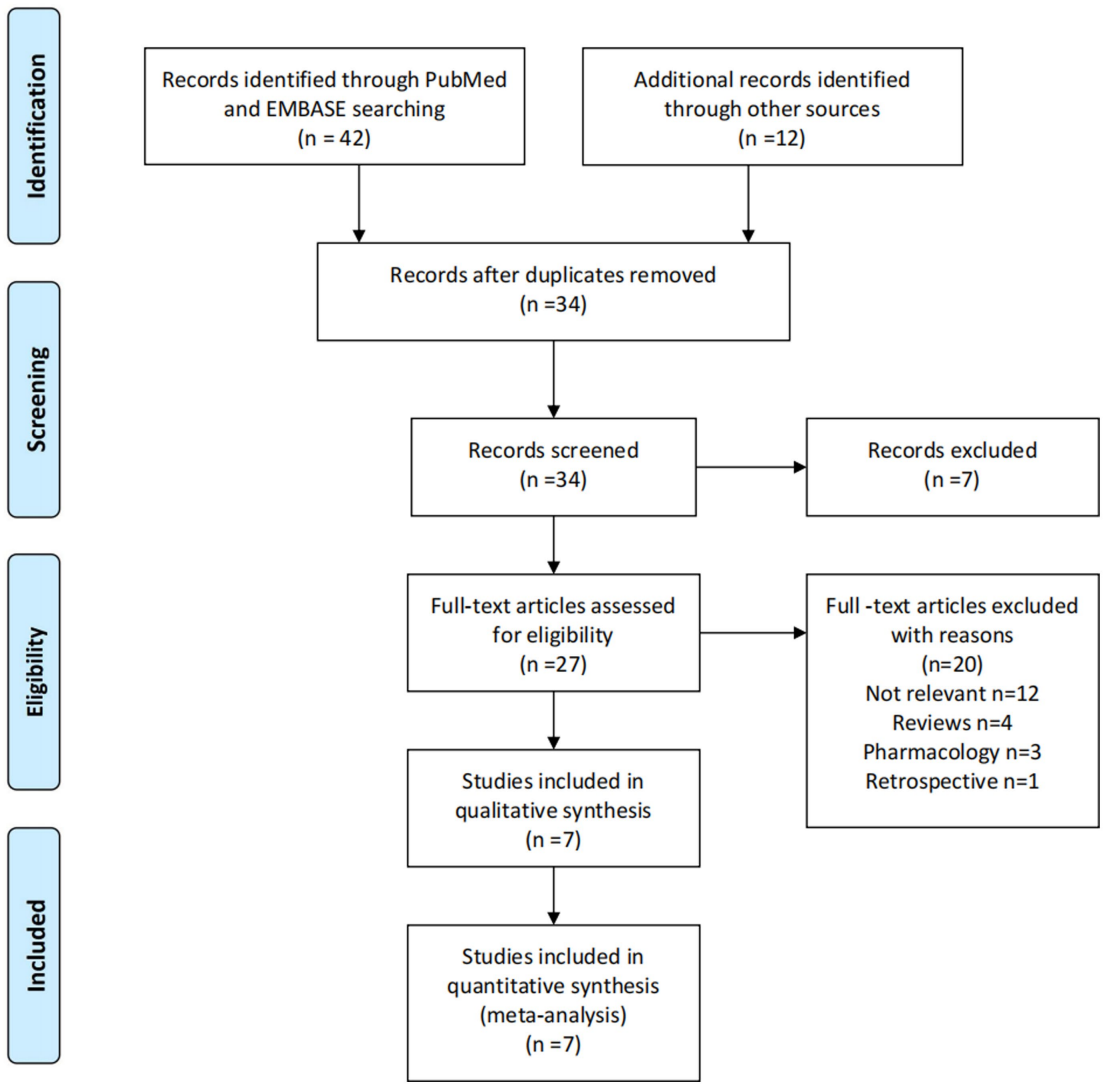
Flowchart outlining the selection process.

Two reviewers (HS and LL) independently screened each article identified through electronic searches for relevance, initially by evaluating the title and abstract, followed by reading the full-text to select articles that met the inclusion criteria. Duplicate articles were removed. A comprehensive record of the selection process was maintained, and a PRISMA flowchart was generated (Figure 1).

### Inclusion and exclusion criteria

Only randomised controlled trials (RCTs) were included in this study. Studies were included if they met all the following criteria. (1) Population: patients diagnosed with ureteral stones who met the criteria of MET. (2) Intervention: the test group received oral mirabegron as MET. (3) Comparators: patients who received other non-mirabegron pharmacological treatments (e.g., diclofenac, tamsulosin, silodosin, etc.) were considered comparators. (4) Outcomes: the primary outcome was the stone expulsion rate (SER). Secondary outcomes included the stone expulsion interval (SEI) and pain episodes during follow-up. The exclusion criteria were as follows. (1) Non-randomised controlled trials (e.g., observational studies, retrospective studies, and case reports). (2) Studies that did not report key outcome measures or those with incomplete or unextractable relevant data. (3) Duplicate publications or studies with overlapping data from other included studies. (4) Reviews, expert opinions, and guidelines.

### Data extraction

An Excel worksheet was designed for data extraction. Two independent researchers (HL and WH) extracted data from all eligible studies and any disagreements were resolved through group discussions. The following information was included in the data collection form: author, publication date, country, sample size, treatment and comparator, study duration, stone location, and stone size.

### Quality assessment

The risk of bias in this study was assessed independently by two authors (LL and HS) using the Cochrane Risk of Bias (RoB) tools 2.0 (19) across several domains, including the randomisation process, deviations from intended interventions, missing outcome data, measurement of the outcome, and selection of the reported result. Any discrepancies between the reviewers were resolved through discussion involving a third investigator (HL). Each domain was assigned a rating of “low,” “some concerns,” or “high.” The overall risk of bias for each trial was determined based on the domain with the highest attributed risk.

### Statistical analysis

The effect size was evaluated using different methods depending on the type of outcome. For dichotomous outcomes, the risk ratio (RR) was computed, while for continuous outcomes, the mean difference (MD) was calculated. Both measures were accompanied by 95% confidence intervals (CIs). Statistical heterogeneity among the included studies was assessed using Cochran’s *Q* test and *I*^2^ statistic, with heterogeneity defined as *I*^2^ > 50% or *p* < 0.05. *τ*^2^ was also calculated to assess the between-study heterogeneity variance. In cases where no heterogeneity was present, a fixed-effects model was used to pool the effect size. Otherwise, a random-effects model was employed.

The robustness of the meta-analysis results was assessed through sensitivity analysis, which aimed to identify the potential impact of individual studies on the overall effect size. Quantitative assessment of publication bias was conducted using funnel plots and Egger’s regression tests. All statistical analyses were carried out using Review Manager software (RevMan, version 5.4.1, Cochrane Collaboration, 2020) and STATA software (Version 14, STATA Corporation, College Station, TX, United States).

## Results

### Characteristics of included studies and quality assessment

Figure 1 illustrates the flowchart outlining the selection process. A total of seven studies, meeting the pre-established inclusion criteria, were selected for the meta-analysis. These studies comprised 361 patients in the mirabegron treatment group and 367 patients in the control group. In all the mirabegron treatment groups, a standardized dosage of 50 mg per day was administered. The follow-up period varied between 4 and 30 days. Table 1 presents a summary of the key characteristics of the included studies.

**Table 1:** Characteristics of included studies.

Author	Year	Country	Study design	Sample size		Intervention		Duration	Stone location	Stone size
				Test	Control	Test	Control			
Bayar (11)	2020	Turkey	RCT	56	Control: 59; silodosin: 54	Mirabegron (50 mg/day)	Control; silodosin 8 mg/day	4 weeks	Proximal, middle and distal ureter	4–10 mm
Chatterjee (12)	2021	India	RCT	50	50	Mirabegron (50 mg/day) + diclofenac (50 mg twice/day for initial 5 days)	Diclofenac 50 mg twice/day for initial 5 days and then on demand	4 weeks	Lower ureter	<10 mm
Seleem (13)	2021	Egypt	RCT (abstract)	37	Control: 40; tamsolosin: 36; tamsolosin + mirabegron: 37	Mirabegron (50 mg/day)	Control; tamsolosin 0.4 mg once daily; tamsolosin 0.4 mg/day + mirabegron 50 mg/day	NA	Distal ureter	5–10 mm
Tang (17)	2021	China	RCT	45	45	Mirabegron (50 mg/day) + tamsolosin 0.2 mg/day	Tamsolosin 0.2 mg/day	4 weeks	Distal ureter	<10 mm
Abdel-Basir (10)	2022	Saudi Arabia	RCT	48	48	Mirabegron (50 mg/day) + ketorolac (30 mg/day for 5 days and then on demand)	Ketorolac of 30 mg/day for 5 days and then on demand	4 weeks	Distal ureter	5–10 mm
Rajpar (16)	2022	Sindh-Pakistan	RCT	100	100	Mirabegron (50 mg/day) + diclofenac 100 mg	Diclofenac 100 mg/day	4 weeks	Distal ureter	<10 mm
Morsy (15)	2022	Egypt	RCT	25	Control: 25; tamsolosin: 25	Mirabegron (50 mg/day) + diclofenac 100 mg (during colic episode)	Tamsolosin 0.4 mg + diclofenac 100 mg (during colic episode); diclofenac 100 mg (during colic episode)	30 days	Distal ureter	<10 mm

**Figure 2:**
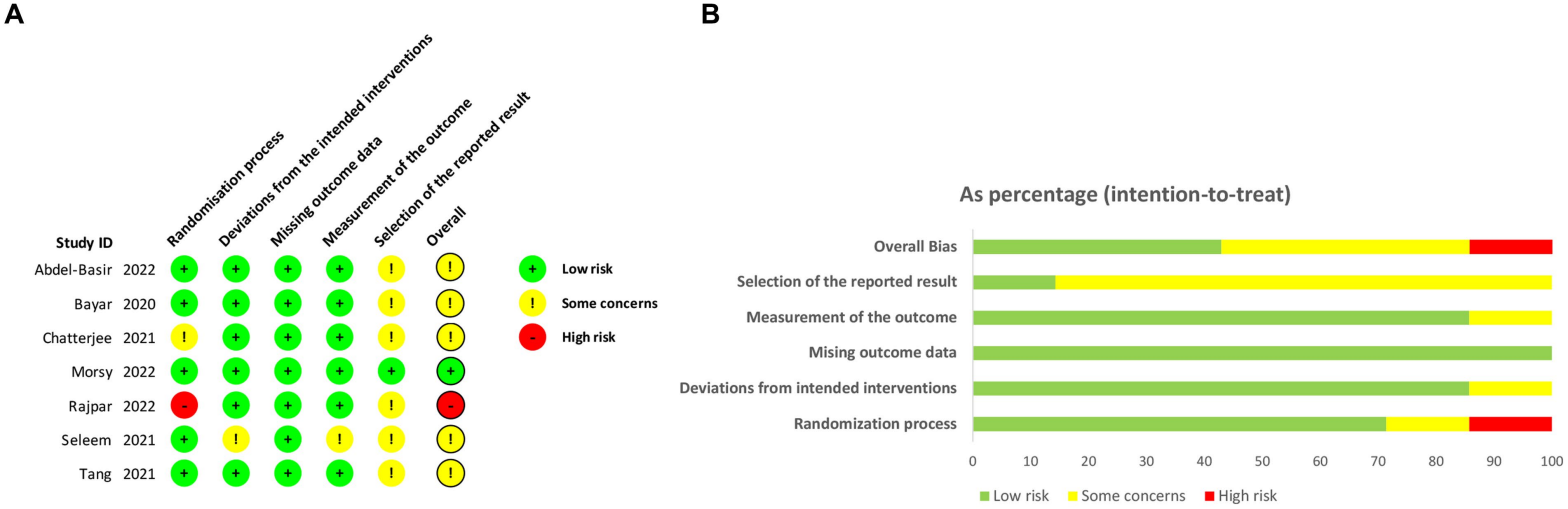
The assessment of risk of bias (RoB). **(A)** Risk of bias domain for each included study; **(B)** Summary of risk of bias assessment.

The results of the assessment of risk of bias (RoB) for each study are presented in Figure 2. Among the included studies, only one was determined to have a high RoB, three studies demonstrated a moderate RoB, and one studies were deemed to have a low RoB. The most frequently observed sources of potential bias in these studies were related to the randomization process and the selection of reported results. A subsequent sensitivity analysis was conducted, which revealed that excluding the high-risk studies did not have a significant impact. As a result, all studies were included in the final analysis.

### SER

**Figure 3:**
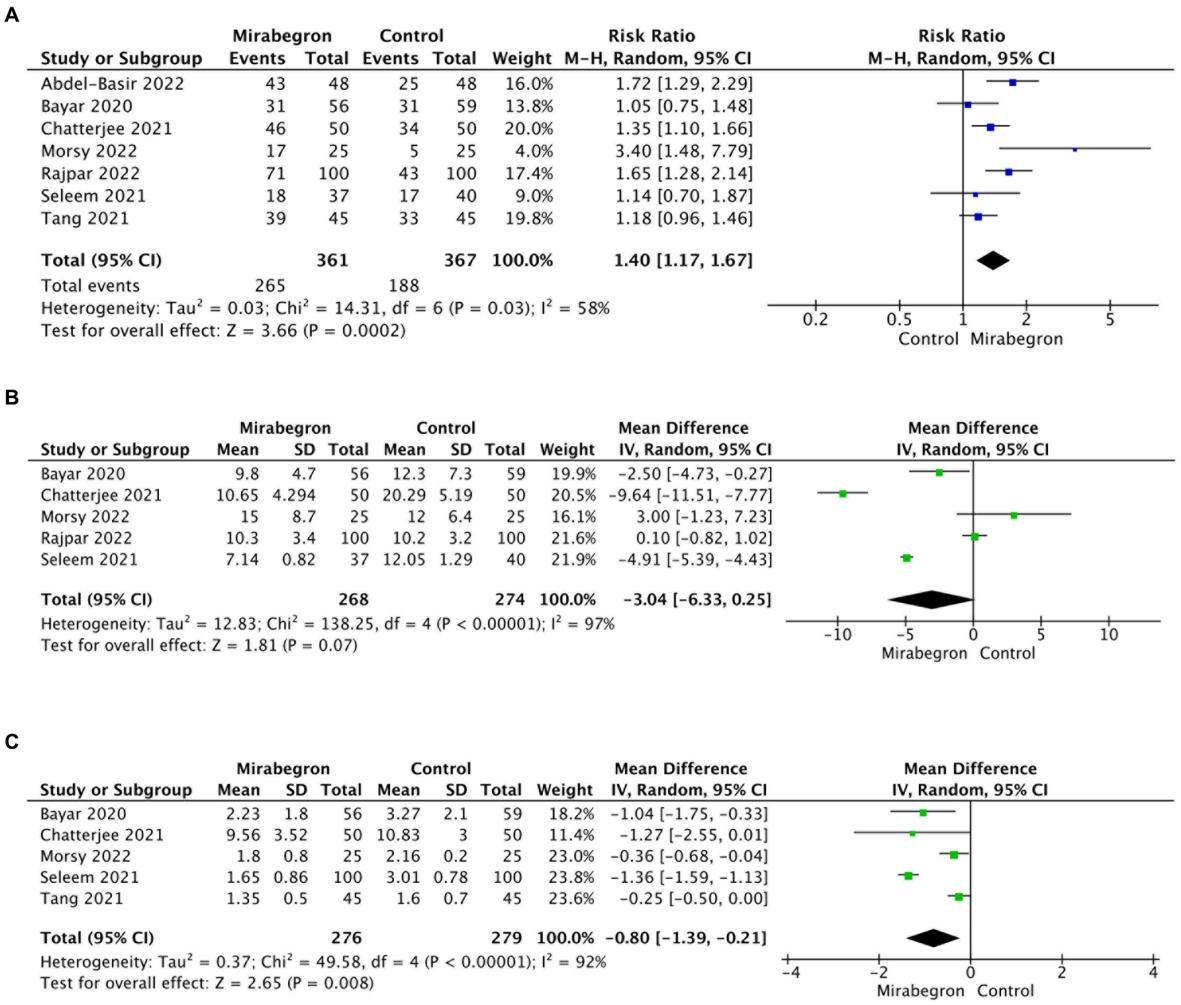
Forest plots showing the pooled results of SER, SEI and pain episodes between mirabegron and control group. **(A)** SER; **(B)** SEI; **(C)** pain episodes. SER, stone expulsion rate; SEI, stone expulsion interval.

A total of seven studies, involving 728 participants (361 in the mirabegron group and 367 in the control group), provided data on the SER for the mirabegron treatment compared to the control group. A random effects model was used to calculate the RR with 95% CI, taking into account a significant heterogeneity (*Q* = 14.31, *p* = 0.03; *I*^2^ = 58%; *τ*^2^ = 0.03). The analysis revealed that mirabegron treatment significantly increased the SER compared to the control group (RR = 1.40; 95% CI =1.17–1.67; *p* < 0.001) (Figure 3A). Further analysis, where each study was excluded sequentially from the analysis and the pooled RR was recalculated, consistently supported the initial findings (Supplementary Figure S1A). This indicates the stability of the meta-analysis results concerning the SER outcomes.

**Figure 4:**
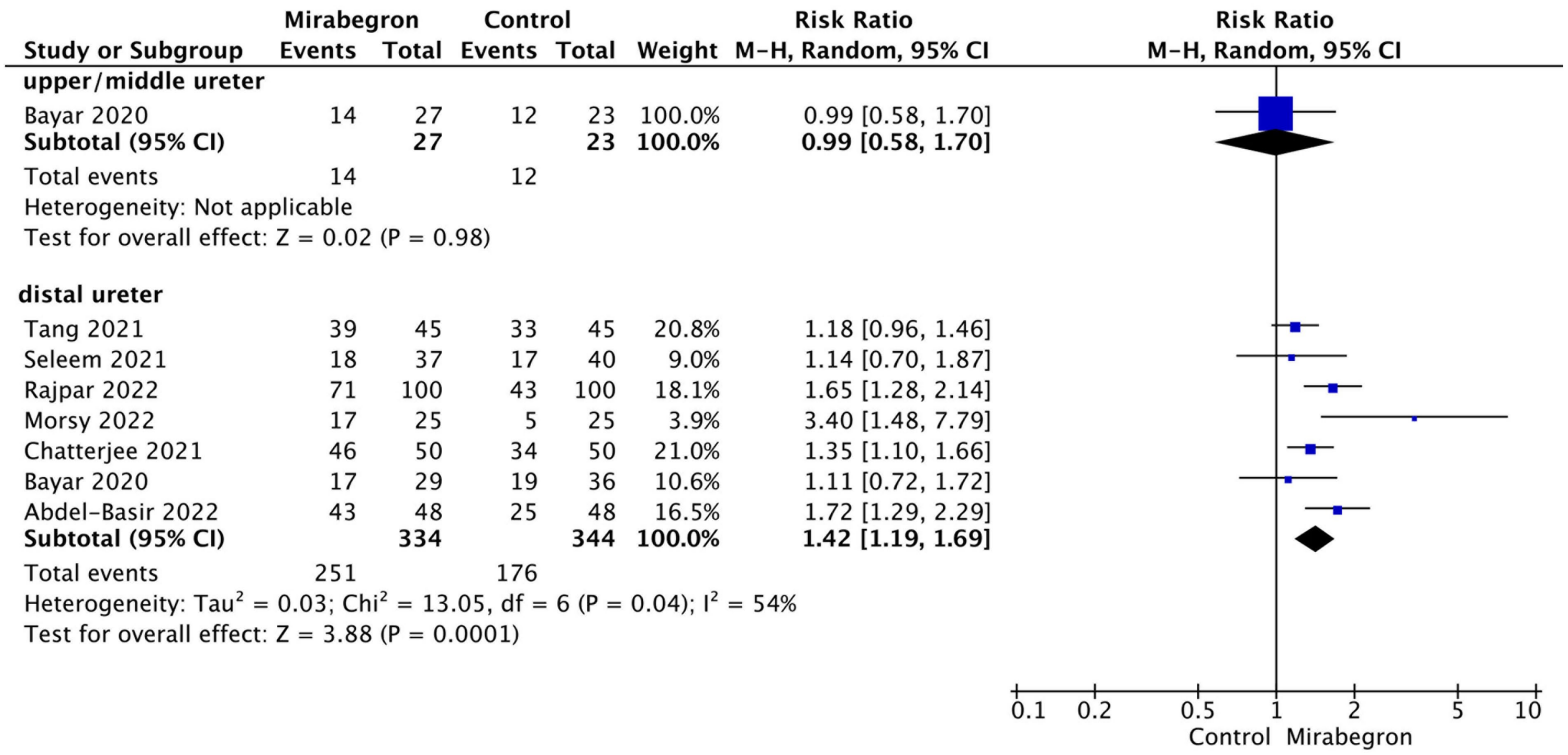
Subgroup analysis based on location of stones.

In order to examine potential variations in the efficacy of mirabegron treatment based on the location of ureteral stones, a subgroup analysis was conducted to compare proximal and mid-ureteral stones with distal ureteral stones. Only one study included data on SER for patients with proximal and mid-ureteral stones. This study reported that mirabegron treatment did not have a significant effect on the SER when compared to the control group (RR = 0.99; 95% CI = 0.58–1.70; *p* = 0.98). In contrast, for patients with distal ureteral stones, the analysis demonstrated a significant increase in the SER with mirabegron treatment compared to the control group (RR = 1.42; 95% CI = 1.19–1.69; *p* < 0.001). Figure 4 presents a forest plot illustrating these findings.

### SEI

A total of five studies, involving 542 cases (268 in the mirabegron group and 274 in the control group), were included in the analysis of SEI. Using a random effects model, the forest plots displayed a pooled mean difference (MD) of −3.04 (95% CI = −6.33 to 0.25; *p* = 0.07) (Figure 3B). There were no significant differences observed in SEI between the two groups. However, the results indicated a high degree of heterogeneity across the studies (*Q* = 138.25, *p* < 0.001; *I*^2^ = 97%; *τ*^2^ = 12.83). Notably, the sensitivity analysis, which systematically excluded each study one by one, produced consistent results with the overall findings (Supplementary Figure S1B), providing evidence of result stability.

### Frequency of pain events during MET

Five articles describing pain episodes including 555 cases (276 in the mirabegron group and 279 in the control group) were included. The pooled MD with 95% CIs was computed using the random effects model because of high heterogeneity (*Q* = 49.58, *p* < 0.001; *I*^2^ = 92%; *τ*^2^ = 0.37). The analysis demonstrated a significant decrease in the frequency of pain events during stone expulsion in the mirabegron group when compared to the control group (MD = −0.80; 95% CI = −0.39 to −0.21; *p* = 0.008). Figure 3C presents a forest plot depicting these results. The sensitivity analysis consistently supported the overall findings, indicating the stability of the results (Supplementary Figure S1C).

### Mirabegron efficacy in MET compared with tamsulosin or silodosin

**Figure 5:**
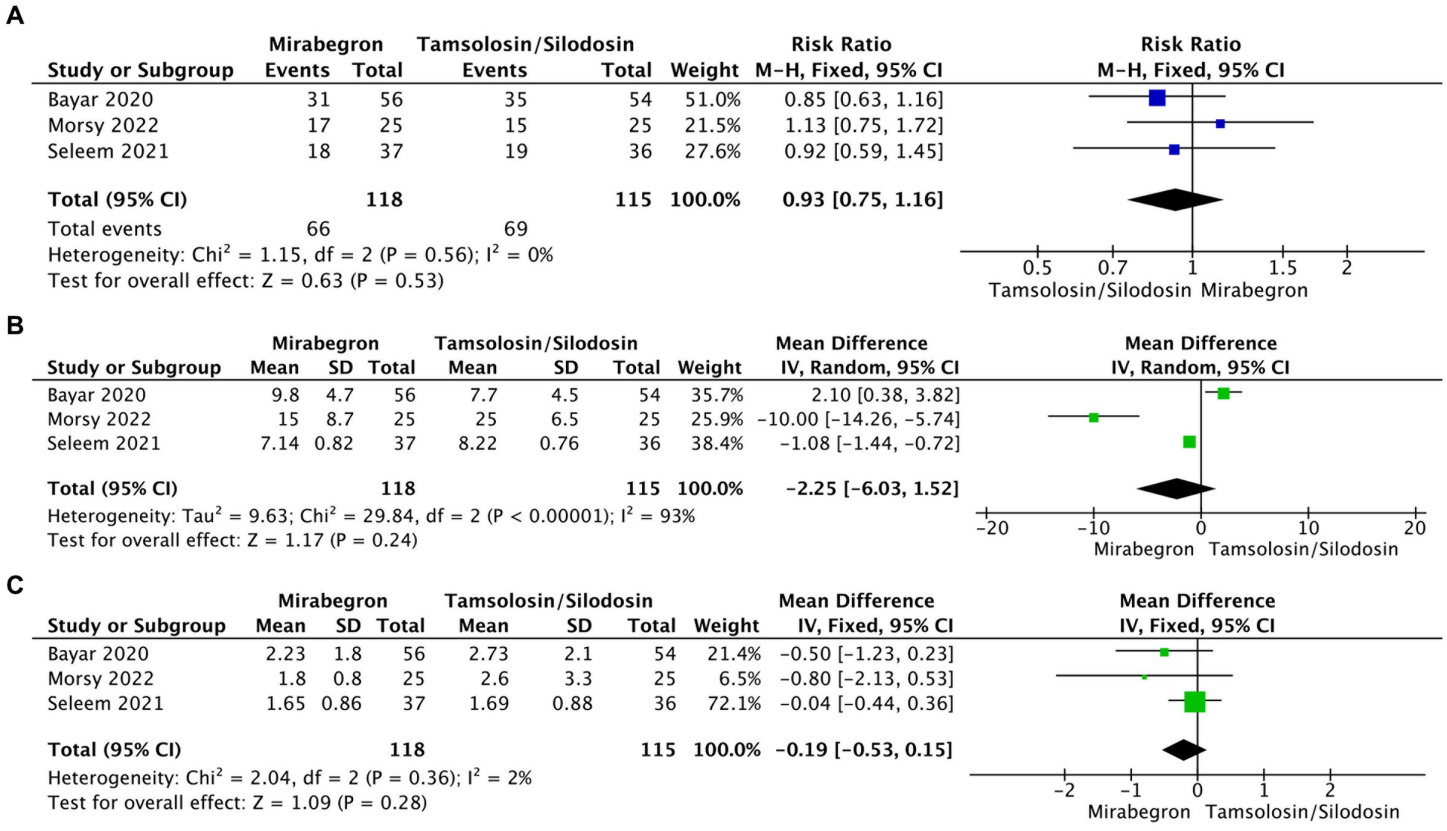
Forest plots showing the pooled results of comparation between mirabegron and tamsulosin/silodosin. **(A)** SER; **(B)** SEI; **(C)** pain episodes. SER, stone expulsion rate; SEI, stone expulsion interval.

To compare the efficacy of mirabegron in MET with α-adrenergic receptor blockers (tamsulosin or silodosin), a total of three studies with relevant data were included, involving 233 patients (118 in the mirabegron group and 115 in the tamsulosin/silodosin group). The pooled RR for SER was 0.93 (95% CI = 0.75–1.16; *p* = 0.53) (Figure 5A), indicating that there was no significant difference in SER between mirabegron and tamsulosin/silodosin treatment. Moreover, the analysis found no significant differences in SEI (MD = −2.25; 95% CI = −6.03 to 1.52; *p* = 0.24) and pain episodes (MD = −0.19; 95% CI = −0.53 to 0.15; *p* = 0.28) between the mirabegron and tamsulosin/silodosin groups (Figures 5B,C).

### Adverse effects

The safety profile of mirabegron for the management of ureteral stone expulsion was investigated. However, owing to the limited data in the included studies, a quantitative evaluation of adverse effects could not be performed. The adverse events reported in the included studies were rare, including hypertension (two cases), nausea and dry mouth (two cases), nasal congestion (one case), constipation (one case), and fever (one case). Among the participants who reported adverse effects, two patients with nausea and dry mouth and two patients with hypertension discontinued the trials. All other adverse effects improved after symptomatic treatment.

### Publication bias

Funnel plots were generated to visually assess publication bias in studies reporting SER, SEI, and pain episodes (Supplementary Figure S2). Egger’s test was used to statistically evaluate publication bias. The funnel plot exhibited symmetrical characteristics, and the *p*-value of Egger’s test was greater than 0.05 for each outcome (*p* = 0.414 for SER; *p* = 0.707 for SEI; *p* = 0.477 for pain episodes), suggesting no significant potential publication bias. In comparison with tamsulosin or silodosin treatment, the *p*-values of Egger’s test were *p* = 0.619 for SER, *p* = 0.902 for SEI, and *p* = 0.477 for pain episodes. The funnel plots are shown in Supplementary Figure S3. No significant publication bias was observed.

## Discussion

Most clinical guidelines suggest that MET should be considered a possible treatment option for distal ureteral stones 5–10 mm in size (3, 4, 20). α-receptor blockers such as tamsulosin are mostly used for MET. In addition, calcium channel antagonists (21), phosphodiesterase type 5 (PDE5) inhibitors (22) and cortisol (23) are reported to be effective in MET. Recently, several studies have shown that mirabegron affects the expulsion of ureteral stones. Solakhan et al. (14) first reported that mirabegron significantly elevated the SER and reduced pain episodes during the expulsion of distal ureteral stones in a retrospective study. A series of randomised controlled trials proved that mirabegron is effective for expulsion, however controversy still exists (12, 15). For example, Bayar et al. (11) reported that mirabegron did not improve SER and had no effect on SEI. Tang et al. (17) demonstrated that mirabegron has a significant impact on improving SER in patients with distal ureteral stones measuring ≤5 mm, while it has no effect on patients with stones measuring >5 mm. Tang et al. (17) showed that mirabegron can play a significant role in improving SER in patients with distal ureteral stones ≤5 mm and no effect in patients with stones > 5 mm. In this meta-analysis, we comprehensively assessed and quantitatively analysed the efficacy and safety of mirabegron in the MET or ureteral stones.

The findings of our study suggest that mirabegron, a β3-adrenergic receptor (β3-AR) agonist, holds promise as a potential treatment for distal ureteral stones measuring <10 mm by effectively increasing SER and reducing the frequency of pain episodes during stone expulsion. β3-AR agonists have been identified as innovative drugs for managing overactive bladder. Matsumoto et al. (6) confirmed the presence of β1-, β2-, and β3-AR expression in the smooth muscle and urothelium of human ureters, including the proximal, middle, and distal segments. Stimulation of β2- and β3-ARs mediates relaxation of the human ureter. They also demonstrated that β3-AR agonists induce concentration-dependent reduction in ureteral muscle contraction. Shen et al. (24) observed a decrease in mRNA and protein expression of β3-AR in the dilated ureter compared to the normal ureter, indicating a potential compensatory mechanism involving increased ureteral contraction to facilitate urine passage through the obstruction. Yalcin et al. (25) found that β-AR agonists inhibit contraction of ureteral smooth muscle and promote ureteral dilation by reducing the frequency of peristalsis in the smooth muscle of the ureter. Thus, mirabegron, functioning as a β3-AR agonist, may promote the expulsion of ureteral stones through this mechanism. However, in the subgroup analysis, mirabegron did not significantly enhance SER for proximal and middle ureter stones. This may be attributed to the inclusion of only one study focusing on proximal and middle ureters in our analysis. Thus, further studies are necessary to determine the effect of mirabegron on proximal and middle ureteral stones.

Our meta-analysis did not reveal a significant difference in SEI between the mirabegron and control groups. However, the results showed a high level of heterogeneity and should be interpreted cautiously, although they were robust after the sensitivity analysis. α-adrenergic receptor blockers such as tamsulosin are currently widely used in MET for ureteral stones. In our meta-analysis, a comparable effect of mirabegron on SER, SEI, and pain episodes compared with tamsulosin or silodosin was observed. Although only three studies were included, the reported adverse effects of mirabegron in this review were relatively rare (1.94%, 7/361 cases) and most were mild. The results indicate mirabegron as a potential alternative option for patients with contraindications to -adrenergic receptor blockers or in cases unresponsive to initial α-blocker treatment.

Our results are consistent with a previous meta-analysis that also focused on this issue (26). The previous meta-analysis comprised only four studies, one of which was a retrospective study (14) and the other concentrated on the use of mirabegron before surgery to enhance the outcomes of semi-rigid ureter lithotripsy (27). We included more updated and higher-quality studies in this meta-analysis and compared the efficacy of mirabegron with tamsulosin or silodosin on MET.

Our study has certain limitations. Firstly, we included only seven studies with just over 361 patients in the mirabegron group; in terms of comparisons with tamsulosin or silodosin treatment, only three studies were included. Secondly, the high level of heterogeneity during the pooling of some endpoints which may have been caused by variations in inclusion and exclusion criteria, sample sizes, and experimental designs, weakened the reliability and stability of the results. Thirdly, the presence of broad confidence intervals in certain included studies, such as those by Bayar and Morsy, contributes to the overall heterogeneity observed in our meta-analysis. This heterogeneity can potentially affect the reliability of our pooled effect size estimates, underscoring the need for a more cautious interpretation of these results. Additionally, due to data limitations in the original literature, we were unable to conduct further subgroup analyses to investigate the sources of heterogeneity. Moreover, most studies were conducted in Asian countries; therefore, the generalisability of the results is limited by regional and ethnic constraints. These limitations suggest that while our findings provide valuable insights into the efficacy of mirabegron for medical expulsive therapy of ureteral stones, they should be interpreted with caution. Future research with more diverse and larger sample sizes, and more consistent study designs, is necessary to validate and extend our findings.

## Conclusion

In general, mirabegron can improve the SER of patients with distal ureteral stones rather than proximal or middle ureteral stones and reduce pain events during stone expulsion but has no effect on the SEI. The effect of mirabegron was comparable to that of tamsulosin and silodosin and has the potential to be a safe alternative treatment for MET for distal ureteral stones. Further high-quality RCTs are required to validate these findings.

## Data availability statement

The original contributions presented in the study are included in the article/Supplementary material, further inquiries can be directed to the corresponding authors.

## Author contributions

HS: Conceptualization, Data curation, Formal analysis, Software, Supervision, Writing – original draft, Writing – review & editing. LL: Data curation, Writing – original draft, Writing – review & editing. HL: Data curation, Formal analysis, Writing – original draft. WH: Formal analysis, Writing – review & editing. GZ: Formal analysis, Supervision, Writing – review & editing. BX: Data curation, Methodology, Writing – review & editing. MF: Conceptualization, Supervision, Writing – original draft, Writing – review & editing. JL: Conceptualization, Project administration, Supervision, Writing – review & editing. YL: Methodology, Validation, Writing – review & editing.

## Supplementary material

SUPPLEMENTARY Figure S1 Sensitive analysis of SER, SEI and pain episodes. A. SER; B. SEI; C. Pain episodes. SER: stone expulsion rate; SEI: stone expulsion interval.

SUPPLEMENTARY Figure S2 Funnel plots showing the publication bias of comparation between mirabegron and control group. A. Forest plots of SER; B. Forest plots of SEI; C. Forest plots of pain episodes. SER: stone expulsion rate; SEI: stone expulsion interval.

SUPPLEMENTARY Figure S3 Funnel plots showing the publication bias of comparation between mirabegron and tamsulosin/silodosin. A. Forest plots of SER; B. Forest plots of SEI; C. Forest plots of pain episodes. SER: stone expulsion rate; SEI: stone expulsion interval.

## References

[1] Raheem OA Khandwala YS Sur RL Ghani KR Denstedt JD. Burden of urolithiasis: trends in prevalence, treatments, and costs. Eur Urol Focus. (2017) 3:18–26. doi: 10.1016/j.euf.2017.04.001, PMID: 28720363

[2] Thongprayoon C Krambeck AE Rule AD. Determining the true burden of kidney stone disease. Nat Rev Nephrol. (2020) 16:736–46. doi: 10.1038/s41581-020-0320-7, PMID: 32753740

[3] Tzelves L Mourmouris P Skolarikos A. Comparison of current guidelines on medical management of stone disease. Arch Esp Urol. (2021) 74:171–82. PMID: 33459633

[4] Türk C Petřík A Sarica K Seitz C Skolarikos A Straub M . EAU guidelines on diagnosis and conservative management of urolithiasis. Eur Urol. (2016) 69:468–74. doi: 10.1016/j.eururo.2015.07.040 26318710

[5] Ordon M Andonian S Blew B Schuler T Chew B Pace KT. CUA guideline: management of ureteral calculi. Can Urol Assoc J. (2015) 9:E837–51. doi: 10.5489/cuaj.3483, PMID: 26788233 PMC4707902

[6] Matsumoto R Otsuka A Suzuki T Shinbo H Mizuno T Kurita Y . Expression and functional role of β3-adrenoceptors in the human ureter. Int J Urol. (2013) 20:1007–14. doi: 10.1111/iju.12093 23360304

[7] Kaya E Sikka SC Oral DY Ozakca I Gur S. β3-adrenoceptor control of lower genitourinary tract organs and function in male: an overview. Curr Drug Targets. (2018) 19:602–12. doi: 10.2174/1389450118666170120165554, PMID: 28117002

[8] Kelleher C Hakimi Z Zur R Siddiqui E Maman K Aballéa S . Efficacy and tolerability of mirabegron compared with antimuscarinic monotherapy or combination therapies for overactive bladder: a systematic review and network meta-analysis. Eur Urol. (2018) 74:324–33. doi: 10.1016/j.eururo.2018.03.020 29699858

[9] Wanajo I Tomiyama Y Yamazaki Y Kojima M Shibata N. Pharmacological characterization of beta-adrenoceptor subtypes mediating relaxation in porcine isolated ureteral smooth muscle. J Urol. (2004) 172:1155–9. doi: 10.1097/01.ju.0000133557.39515.b6 15311061

[10] Abdel-Basir Sayed M Moeen AM Saada H Nassir A Tayib A Gadelkareem RA. Mirabegron as a medical expulsive therapy for 5–10 mm distal ureteral stones: a prospective, randomized, comparative study. Turk J Urol. (2022) 48:209–14. doi: 10.5152/tud.2022.22014, PMID: 35634939 PMC9730259

[11] Bayar G Yavuz A Cakmak S Ofluoglu Y Kilinc MF Kucuk E . Efficacy of silodosin or mirabegron in medical expulsive therapy for ureteral stones: a prospective, randomized-controlled study. Int Urol Nephrol. (2020) 52:835–40. doi: 10.1007/s11255-019-02368-y 31873859

[12] Chatterjee S Jalan V Pal DK. An observational study on the efficacy of mirabegron in medical expulsive therapy of the lower ureteric calculus. Urol Sci. (2021) 32:132–6. doi: 10.4103/UROS.UROS_19_21

[13] Seleem M Abd Elwahab K Sakr A Ali M Desoky E. Mirabegron, tamsulosin monotherapy versus combination in treatment of distal ureteric stone. A randomized controlled clinical trial. Eur Urol. (2021) 79:S370. doi: 10.1016/S0302-2838(21)00646-1

[14] Solakhan M Bayrak O Bulut E. Efficacy of mirabegron in medical expulsive therapy. Urolithiasis. (2019) 47:303–7. doi: 10.1007/s00240-018-1075-5, PMID: 30078089

[15] Morsy S Nasser I Aboulela W Abdelazim MS Ali H. Efficacy of mirabegron as medical expulsive therapy for distal ureteral stones: a prospective, randomized, double-blinded, controlled study. Urol Int. (2022) 106:1265–71. doi: 10.1159/000521171, PMID: 35100594

[16] Rajpar ZH Memon II Soomro KQ Hussain SA Mughal SA Soomro N. Comparison of the efficacy of medical expulsive therapy for the treatment of distal ureteric stones with and without mirabegron. J Liaquat Univ Med Health Sci. (2022) 21:11–5. doi: 10.22442/jlumhs.2021.00745

[17] Tang QL Wang DJ Zhou S Tao RZ. Mirabegron in medical expulsive therapy for distal ureteral stones: a prospective, randomized, controlled study. World J Urol. (2021) 39:4465–70. doi: 10.1007/s00345-021-03772-9, PMID: 34241685

[18] Page MJ McKenzie JE Bossuyt PM Boutron I Hoffmann TC Mulrow CD . The PRISMA 2020 statement: an updated guideline for reporting systematic reviews. Syst Rev. (2021) 10:89. doi: 10.1186/s13643-021-01626-4 33781348 PMC8008539

[19] Higgins JP Altman DG Gøtzsche PC Jüni P Moher D Oxman AD . The Cochrane Collaboration’s tool for assessing risk of bias in randomised trials. BMJ. (2011) 343:d5928. doi: 10.1136/bmj.d5928, PMID: 22008217 PMC3196245

[20] Türk C Petřík A Sarica K Seitz C Skolarikos A Straub M . EAU guidelines on interventional treatment for urolithiasis. Eur Urol. (2016) 69:475–82. doi: 10.1016/j.eururo.2015.07.041 26344917

[21] Ye Z Yang H Li H Zhang X Deng Y Zeng G . A multicentre, prospective, randomized trial: comparative efficacy of tamsulosin and nifedipine in medical expulsive therapy for distal ureteric stones with renal colic. BJU Int. (2011) 108:276–9. doi: 10.1111/j.1464-410X.2010.09801.x, PMID: 21083640

[22] Bai Y Yang Y Wang X Tang Y Han P Wang J. Tadalafil facilitates the distal ureteral stone expulsion: a meta-analysis. J Endourol. (2017) 31:557–63. doi: 10.1089/end.2016.0837 28384011

[23] Sridharan K Sivaramakrishnan G. Medical expulsive therapy in urolithiasis: a mixed treatment comparison network meta-analysis of randomized controlled clinical trials. Expert Opin Pharmacother. (2017) 18:1421–31. doi: 10.1080/14656566.2017.1362393, PMID: 28756724

[24] Shen H Chen Z Mokhtar AD Bi X Wu G Gong S . Expression of beta-adrenergic receptor subtypes in human normal and dilated ureter. Int Urol Nephrol. (2017) 49:1771–8. doi: 10.1007/s11255-017-1667-y, PMID: 28756611

[25] Yalcin S Ertunc M Ardicli B Kabakus IM Tas TS Sara Y . Ureterovesical junction obstruction causes increment in smooth muscle contractility, and cholinergic and adrenergic activity in distal ureter of rabbits. J Pediatr Surg. (2013) 48:1954–61. doi: 10.1016/j.jpedsurg.2013.01.030 24074674

[26] Cai D Wei G Wu P Huang Y Che X Zhang Y . The efficacy of mirabegron in medical expulsive therapy for ureteral stones: a systematic review and meta-analysis. Int J Clin Pract. (2022) 2022:1–7. doi: 10.1155/2022/2293182 PMC9159211 35685505

[27] Bayar G Kilinc MF Yavuz A Aydın M. Adjunction of tamsulosin or mirabegron before semi-rigid ureterolithotripsy improves outcomes: prospective, randomized single-blind study. Int Urol Nephrol. (2019) 51:931–6. doi: 10.1007/s11255-019-02142-0, PMID: 30989563

